# Antimicrobial Activity of Catechol-Containing Biopolymer Poly[3-(3,4-dihydroxyphenyl)glyceric Acid] from Different Medicinal Plants of Boraginaceae Family

**DOI:** 10.3390/antibiotics12020285

**Published:** 2023-02-01

**Authors:** Vakhtang Barbakadze, Maia Merlani, Lali Gogilashvili, Lela Amiranashvili, Anthi Petrou, Athina Geronikaki, Ana Ćirić, Jasmina Glamočlija, Marina Soković

**Affiliations:** 1 TSMU I. Kutateladze Institute of Pharmacochemistry, Tbilisi 0159, Georgia; 2 School of Pharmacy, Aristotle University, 54124 Thessaloniki, Greece; 3 Mycological Laboratory, Institute for Biological Research “Siniša Stanković”–National Institute of Republic of Serbia, University of Belgrade, Blvd. Despot Stefan 142, 11000 Belgrade, Serbia

**Keywords:** extracts, catechol-based biopolymers, antimicrobial activity, resistant strains, Boraginaceae family

## Abstract

This study reports the antimicrobial activities of the biopolymers poly[3-(3,4-dihydoxyphenyl)glyceric acid] (PDHPGA) and poly[2-methoxycarbonyl-3-(3,4-dihydroxyphenyl)oxirane] (PMDHPO), extracted from the six plants of Boraginaceae family: *Symphytum asperum* (***SA***), *S. caucasicum* (***SC***), *S. gr* and *iflorum* (***SG***), *Anchusa italica* (***AI***), *Cynoglosum officinale* (***CO***), and *Borago officinalis* (***BO***) collected in various parts of Georgia. The study revealed that the antibacterial activities were moderate, and biopolymers from only three plants showed activities against all tested bacteria. Biopolymers from ***CO*** stems as well as ***SC*** and ***AI*** did not show any activity except low activity against a resistant *P. aeruginosa* strain, which was the most resistant among all three resistant strains. On the other hand, the antifungal activity was better compared to the antibacterial activity. Biopolymers from ***BO*** stems exhibited the best activities with MIC/MFC at 0.37–1.00 mg/mL and 0.75–1.5 mg/L, respectively, followed by those from ***SG*** stems. Biopolymers from ***SC*** and ***AI*** roots showed antifungal activities against all six fungi, in contrast to the antibacterial activity, while biopolymers from ***CO*** stems and ***SA*** roots had activities against four fungi and one fungus, respectively. The sugar-based catechol-containing biopolymers from ***BO*** stems demonstrated the best activities among all tested biopolymers against *T. viride*, *P. funiculosum*, *P. cyclpoium var verucosum*, and *C. albicans* (MIC 0.37 mg/mL). In addition, biopolymers from ***SG*** stems were half as active against *A. fumigatus* and *T. viride* as ketoconazole. Biopolymers from all plant materials except for ***CO*** stems showed higher potency than ketoconazole against *T. viride.* For the first time, it was shown that all plant materials exhibited better activity against *C. albicans*, one of the most dreadful fungal species.

## 1. Introduction

Biopolymers are chain-like molecules that consist of repeated chemical blocks derived from renewable resources that may decompose in the environment. The recent popular use of biomaterials is due to the fact that reducing the usage of non-renewable resources reduces the environmental pollution produced by synthetic materials. Furthermore, biopolymers have attracted significant attention as a class of polymer materials with a wide range of applications, especially in the medical [1] and pharmaceutical fields [2,3]. Due to their biodegradability and non-toxic nature, biopolymers play a positive role in the maintenance of a clean and safe environment.

Hydroxycinnamic acids have been extensively used in the preparation of polyesters, but there are also reports about their use in the synthesis of polyamides and poly(anhydride esters), among many others. These polymers have a wide spectrum of applications, ranging from industrial (e.g., (super) engineered plastics) to biomedical domains (e.g., drug delivery systems and shape memory materials) [4]. Plant-derived, adhesive polymers from natural sources such as 3,4-dihydroxycinnamic acid (caffeic acid) and 4-hydroxycinnamic acid (*p*-coumaric acid) modified by transesterification were found to show strong adhesive characteristics on metal surfaces, which were equivalent to conventional superglues from petroleum resources [5]. Lignin-mimetic polyesters prepared using an in-bulk polycondensation of bioavailable aromatic hydroxyl(carboxylic acids) can be applied as an environmentally degradable plastic with extremely high performance and can be used in automobiles, aircraft, electronic devices, and other materials [6].

Phenols, phenolic-rich plant extracts, including catechol and catechol-based polymers, exhibiting antibacterial and antifungal activities are of great interest to researchers and industry [7]. Antimicrobial polymers can slow or inhibit the growth of drug-resistant strains and present high antimicrobial efficacy due to the various antimicrobial modes and polymeric structures [8]. The phenolic-rich plant extracts of the Boraginaceae family have important therapeutic (antimicrobial, antitumor, antiviral, anti-inflammatory, cardiotonic, contraceptive, antiplatelet activity, etc.) and cosmetic applications [9]. Their pharmacological effects are related to the presence of various classes of compounds such as phenolic acids, phenols, naphthoquinones, flavonoids, terpenoids, and the purine derivative allantoin. However, plants of the Boraginaceae family are also rich in hepatotoxic pyrrolizidine alkaloids (which serve as chemical defense compounds, mainly against herbivores), and thus their consumption is restricted; nevertheless, the use of Boraginaceae plants as poultices for wounds does not raise any objection [9,10]. There are many references in the literature regarding the antimicrobial activity of phenolic plant extracts. Thus, Raphaelli et al. [11] reported an evaluation of the antimicrobial activities of apple phenolic extracts against *Escherichia coli*, *Listeria monocytogenes*, *Salmonella* Typhimurium, and *Staphylococcus aureus.* Hussain et al. [12] studied the composition and antimicrobial activities of different Emirati date (*Phoenix dactylifera* L.) pits against *Staphylococcus aureus* (ATCC 29123), *Escherichia coli* (ATCC 25922), and *Candida albicans* (ATCC 66027). Different extracts from these six date pits showed different antimicrobial activities. The only common finding was that none exhibited activity against *C. albicans*. Dikpınar et al. [13], in his review, mentioned the isolation of numerous phenolic compounds from plants/plant extracts that showed the ability to prevent the growth of bacteria and fungi. The review revealed that phenolic compounds from medicinal plants demonstrated different antimicrobial activities according to their molecular structures, such as the permeabilization and destabilization of the plasma membrane or the inhibition of extracellular enzymes. Salazar-Aranda [14] reported an antimicrobial activity evaluation of extracts from 17 plants from different regions of northeast Mexico against three Gram-negative bacterial strains (*Pseudomonas aeruginosa*, *Klebsiella pneumoniae*, and *Acinetobacter baumannii*), three Gram-positive bacterial strains (*Enterococcus faecalis* and two *Staphylococcus aureus* strains), and seven clinically isolated yeasts (*Candida albicans*, *C. krusei*, *C. tropicalis*, *C. parapsilosis*, and *C. glabrata*). It was shown that the antimicrobial activities from different plants and against different bacterial and fungal strains ranged from 31.25 to 250 mg/mL. In addition, plant phenolics and their extracts can be very good inhibitors of many foodborne pathogenic and spoilage bacteria [15,16]. In an extensive review, Taco et al. [17] reported the antimicrobial activities of plant extracts from several sources such as grape pomace, grape seed, apple, and various exotic fruit and medicinal plant samples. It was mentioned that red grape pomace exhibited a strong bactericidal effect against *E. coli* and *S. aureus* at 12 mg/mL [18]. Grape seed extracts also appeared to be active against bacteria such as *S.* Typhimurium, *Listeria monocytogenes*, *Bacillus* spp., *Pseudomonas aeruginosa*, and *Campylobacter* spp. [19,20], while red pitahaya (exotic fruits extracts) demonstrated a good antimicrobial spectrum against Gram-positive and Gram-negative bacteria, yeasts, and molds at concentrations from 7.8 μg/mL to 50 mg/mL [21,22].

Recently, the antimicrobial properties of some caffeic and 3-(3,4-dihydroxyphenyl)glyceric acid derivatives against Gram-positive and Gram-negative bacteria, a series of fungi [23], and three resistant bacterial strains (methicillin-resistant *Staphylococcus aureus*, resistant *Escherichia coli*, and resistant *Pseudomonas aeruginosa)* were evaluated in vitro [24]. The studies revealed that some caffeic and 3-(3,4-dihydroxyphenyl)glyceric acid derivatives appeared to be more active than the reference drugs ampicillin and streptomycin, whereas the antifungal activities of all compounds were greater than the reference drug ketoconazole, while some compounds appeared to be more active than bifonazole [23]. Kepa et al. [25] studied the antibacterial potential of caffeic acid (CA) alone and in an antibiotic–phytochemical combination against *Staphylococcus aureus* reference and clinical strains isolated from infected wounds. The effects of caffeic acid on the *S. aureus* strains were different, with variations in the minimum inhibitory concentration (MIC) from 256 μg/mL to 1024 μg/mL. The study revealed that the antimicrobial action of CA is probably strain-dependent. On the other hand, it was postulated that the antimicrobial activity of CA against *S. aureus* clinical isolates is higher than that of the reference drug. Furthermore, CA is capable of increasing the antimicrobial effect in combination with antibiotics.

**Figure 1:**
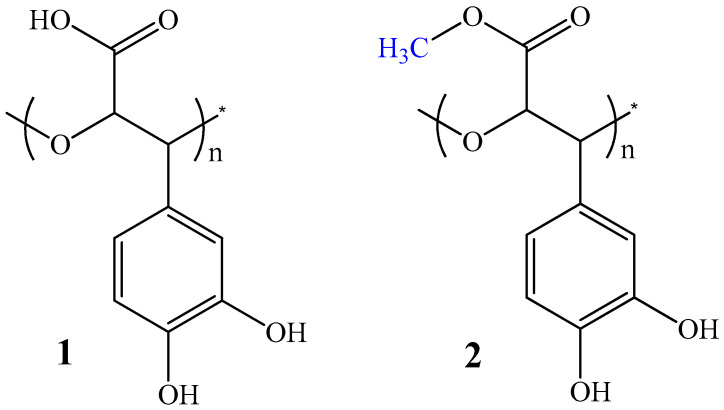
Poly[3-(3,4-dihydroxyphenyl)glyceric acid] (PDHPGA) (**1**) and poly[2-methoxycarbonyl-3-(3,4-dihydroxyphenyl)oxirane] (PMDHPO) (**2**).

In our previous studies, we reported the isolation of water-soluble mucilaginous high-molecular-weight (>1000 kDa or 500 kDa) fractions (HMFs) from plants of the Boraginaceae family: *Symphytum asperum* (***SA***), *S. caucasicum* (***SC***), *S. gr* and *iflorum* (***SG***), *S. officinale* (***SO***), *Anchusa italica* (***AI***), *Cynoglosum officinale* (***CO***), *Borago officinalis* (***BO***), and *Paracynoglossum imeretinum* (***PI***) through the ultrafiltration of hot-water extracts using membrane filters with a cut-off values of 1000 kDa or 500 kDa. The HMFs obtained using this procedure are free of pyrrolizidine alkaloids and allantoin. According to UV data , IR spectra, and different techniques of NMR spectroscopy (^13^C NMR, APT, ^1^H NMR, 1D NOE, 2D ^1^H/^13^C HSQC, and 2D DOSY) [26,27] (Supplementary materials, Figures S1–S8, Table S1), the main chemical constituent of the HMFs of the above-mentioned medicinal plants was found to be an exotic bioactive carbohydrate-based catechol-containing bio-polyether, namely poly[3-(3,4-dihydroxyphenyl)glyceric acid] (PDHPGA) (**1**, Figure 1), that is, poly[oxy-1-carboxy-2-(3,4-dihydroxyphenyl)ethylene] (POCDPE). The repeating unit of PDHPGA is a 3-(3,4-dihydroxyphenyl)glyceric acid residue. Most of the carboxyl groups of PDHPGA from ***AI***, ***SG***, and ***BO***, unlike of biopolymers from ***SA***, ***SC***, ***SO***, ***CO***, and ***PI***, are methyl-esterified and are named poly[2-methoxycarbonyl-3-(3,4-dihydroxyphenyl)oxirane] (PMDHPO) (**2** in Figure 1) [26].

The polyoxyethylene (polyethylene glycol) chain is the backbone of polymeric molecule PDHPGA. 3,4-Dihydroxyphenyl (catechol) and carboxyl groups are regular substituents at two carbon atoms in the chain of PDHPGA [26]. On the other hand, a poly (2,3-glyceric acid ether) chain is the backbone of the polymeric molecule of PDHPGA. The basic moiety of poly (2,3-glyceric acid ether) is glyceric acid, a natural three-carbon sugar acid, which is an oxidative form of the simplest of all common aldoses, namely glyceraldehyde. In this case, a poly (2,3-glyceric acid ether) chain constitutes the backbone of the polymeric molecule of PDHPGA, with catechol groups as the regular substituents in the chain. PDHPGA, bearing a lot of catecholic groups along the polymeric chain, is a novel sugar-based multicatechol-containing biopolymer. Thus, we can consider PDHPGA as a representative of an exotic class of carbohydrate-based catechol-functionalized biopolymers with ether bonds. Such biopolymers, to our knowledge, are unknown in nature, as all above-mentioned synthetic and natural polymers are either polyesters or copolymers [4,5,6].

PDHPGA exhibits high immunomodulatory (anticomplementary), antioxidant, anti-inflammatory, wound and burn healing, and anticancer activities, both in vitro and in vivo [27].

Later, the antibacterial properties of natural polyethers from different species of the Boraginaceae family (PDHPGA-SA, PDHPGA-SC, PDHPGA-SG, PDHPGA-AI, and PDHPGA-CO), along with the synthetic polymers poly[2-methoxycarbonyl-3-(3,4-dimethoxyphenyl)oxirane (PMDMPO), poly[2-methoxycarbonyl-3-(3,4-dibenzyloxyphenyl)oxirane] (PMDBPO), and poly[2-methoxycarbonyl-3-(3,4-dihidroxphenyl)oxirane] (PMDHPO), were investigated against the pathogenic strains *S. aureus* ATCC 25923 and *E. coli* ATCC 25922. It was shown that only a synthetic oligomeric analogue of PMDHPO exhibited antimicrobial activity against these pathogenic strains at the concentrations that were used [27]. Interesting data obtained about the antimicrobial activities of synthetic compounds prompted us to continue research in this field and examine natural polyethers from the above-mentioned species of the Boraginaceae family against Gram-positive and Gram-negative bacteria, three resistant bacterial strains (MRSA, *E. coli*, and *P. aeruginosa*), and a panel of fungi.

The aim of this study was to examine and evaluate the antimicrobial activities of sugar-based catechol-containing biopolymers from ***SA***, ***SC***, ***SG***, ***AI***, ***CO*,** and ***BO***.

## 2. Results and Discussion

### 2.1. Chemistry

According to data from UV, IR, liquid-state ^1^H, ^13^C NMR, gCOSY, and 2D heteronuclear ^1^H/^13^C gHSQCED experiments, the main chemical constituent of the water-soluble high-molecular fractions from the stems and roots of ***SA***, ***SC***, ***SG***, ***SO***, ***AI***, ***CO***, and ***BO*** was found to be the biologically active caffeic-acid-derived polymer PDHPGA [26]. Most of the carboxyl groups of PDHPGA from ***AI**, **SG***, and ***BO***, unlike of biopolymers from ***SA, SC, SO***, ***CO***, and ***PI***, are methyl-esterified [27].

Tests of the fractionation and molecular weight distribution of a biologically active PDHPGA preparation from ***SA*** were carried out. The molecular weights and contents of different fractions for the PDHPGA preparation were simultaneously determined using high-performance size-exclusion chromatography (HPSEC) coupled with multiangle laser light scattering (MALLS) and a refractive index detector (RID) with a refractive index increment (dn/dc) [28].

Absorption maxima at 212 nm, 236 nm (shoulder), 282 nm (shoulder), and 286 nm were observed in the UV spectra (H_2_O, λ_max_, nm) of PDHPGA-SA, PDHPGA-SC, PDHPGA-SG, PDHPGA-AI, PDHPGA-CO, and PDHPGA-BO, which could be attributable to substituted phenols (Figure S1). The IR spectra of PDHPGA-SA, PDHPGA-SC, PDHPGA-SG, PDHPGA-AI, PDHPGA-CO, and PDHPGA-BO contained absorption bands characteristic of phenol-carboxylic acids: 3425 (OH); 2924 (CH); 1605 (ionized carboxyl) and 1736 cm^−1^ for its ester form; 1512 and 1443 (aromatic C=C); 1404 and 1219 (phenols); 1265, 1080, and 1018 (R-O-R’); 872 (C-H in the aromatic ring with one isolated hydrogen atom); and 818 cm^−1^ (C-H in the aromatic ring with two neighboring hydrogen atoms (Figure S2). The signal assignments of the ^13^C and ^1^H NMR spectra of PDHPGA-SA, PDHPGA-SC, and PDHPGA-CO and of the methylated carboxylic groups of PDHPGA-SG, PDHPGA-AI, and PDHPGA-BO are given in Supplementary Materials Table S1.

**Figure 2:**
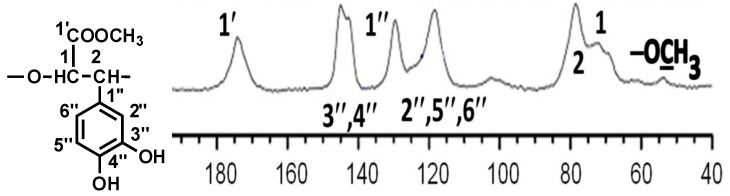
Solid-state ^13^C NMR spectrum of PMDHPO. Solid-state ^13^C{^1^H} CP/MAS NMR spectrum, recorded at 9.4 T employing magic-angle spinning at 14.0 kHz and a CP time of 3.0 ms, showing the repeating unit δ (13C) of PMDHPO (ppm).

The 2D DOSY experiment gave similar diffusion coefficients for the methylated and non-methylated signals of PDHPGA. Both sets of signals fell in the same horizontal range (Figure S8). This implies similar (same order of magnitude) molecular weights for methylated and non-methylated polymers. Thus, we concluded that the NMR signals of both the methylated and non-methylated carboxylic groups originated from the same PDHPGA polymer. In order to confirm the proposed structure of the repeating unit of non-methylated PDHPGA and the mostly methylated carboxylic groups of these poorly water-soluble high-molecular-weight PMDHPOs, the ^13^C solid-state NMR spectrum was recorded (Figure 2), and total assignments of the signals are given in Table 1. The total assignments of the signals of the solid state ^13^C NMR of a mixture of PDHPGA + PMDHPO were in good agreement with the results of the above-mentioned liquid-state ^13^C NMR classical spectra of the PDHPGA and PMDHPO samples in D_2_O.

Thus, according to data from different techniques of NMR spectroscopy, the main chemical constituent of the high-molecular-weight fractions from the medicinal plants ***SA**, **SC, SG, AI, CO***, and ***BO*** is poly[3-(3,4-dihydroxyphenyl)glyceric acid] (PDHPGA), which is poly[oxy-1-carboxy-2-(3,4-dihydroxyphenyl)ethylene] (POCDPE).

### 2.2. Biological Evaluation

#### 2.2.1. Antibacterial Activity Evaluation

The sugar-based catechol-containing biopolymers from ***SA***, ***SC***, ***SG***, ***AI***, ***CO***, and ***BO*** were tested for their antibacterial activities against a panel of six bacterial species, including *Staphylococcus aureus*, *Escherichia coli*, and *Pseudomonas aeruginosa*, using a microdilution method for the determination of the minimal inhibitory (MIC) and minimal bactericidal concentrations (MBC). The activities of these polymers were moderate to low, with MICs in range of 0.75–6.00 mg/mL and MBCs of 1.00–9.00 mg/mL (Table 2). As can be seen in Table 2, the polymers from ***SG*** stems exhibited the best activities among all tested compounds, with MIC values of 0.75 mg/mL, while the polymer from ***SA*** roots showed the lowest activity (MIC 6.00 mg/mL) compared to ampicillin and streptomycin, which were used as reference drugs. Moderate activity was also observed against *E. coli* and *P. aeruginosa* for polymers from ***BO*** stems (MIC 1.00 mg/mL). On the other hand, the sugar-based catechol-containing biopolymers from ***SC*** and ***AI*** roots and ***CO*** stems did not show any activity against the bacteria in this experiment except for ***AI*** roots against *E. coli*. Thus, the order of activity can be presented as follows: ***SG*** stems > ***BO*** stems > ***SA*** roots. The bacteria most sensitive to these biopolymers appeared to be *E. coli*, while *S. aureus* was the most resistant.

These biopolymers were tested against resistant strains of the mentioned bacteria. The best activity was observed against res. *P. aeruginosa*, expressed by biopolymers from ***BO*** and ***SG*** stems (MIC/MBC at 0.75 mg/mL). The latter showed better activity against MRSA and res. *E. coli*, with MIC 1.00 mg/mL and 0.75 mg/mL, respectively, than the biopolymers from the materials of the two other plants. The order of activity against resistant strains can be presented as follows: MRSA > res. *P. aeruginosa* > res. *E. coli*.

#### 2.2.2. Antifungal Activity Evaluation

These biopolymers were also evaluated for their antifungal activities against six fungal species, and the results are presented in Table 3. The sugar-based catechol-containing biopolymers exhibited better antifungal activities compared to antibacterial activities, with MICs in the range of 0.37–6.00 mg/mL and MFCs of 0.75–9.00 mg/mL. The best activity was observed for biopolymers from ***BO*** stems, with MIC/MFC at 0.37–1.00 mg/mL and 0.75–1.5 mg/L, respectively, followed by those from ***SG*** stems. It should be mentioned that biopolymers from ***SC*** and ***AI*** roots showed antifungal activities against all six fungi, while those from ***CO*** stems and ***SA*** showed antifungal activities against four fungi and one fungus, respectively, in contrast to the antibacterial activity. In general, the order of activity can be presented as: ***BO*** stems > ***SG*** stems > ***SC*** roots > ***AI*** roots > ***CO*** stems > ***SA*** roots. The best activity among all tested biopolymers was found for the biopolymer from ***BO*** stems against *Trichoderma viride*, *Penicillium funicolosum*, *Penicillium verrucosum var. cyclopium*, and *Candida albicans* (MIC 0.37 mg/mL). The same good activity was shown by biopolymers from ***SG*** stems against *Aspergillus fumigatus* and *T. viride*, which was half as active as ketoconazole, the reference drug. It should be mentioned that all sugar-based catechol-containing biopolymers from all plant material except for ***CO*** stems were more potent than ketoconazole against *T. viride*. The most sensitive fungus to these biopolymers appeared to be *T. viride*, while *P. funiculosum* was the most resistant, followed by *P. verrucosum var. cyclopium.* It should be noticed that, in general, sugar-based catechol-containing biopolymers demonstrated quite good activity against *C. albicans* in comparison to other strains except *T. viride*.

Antimicrobial studies revealed that PDHPGA from ***BO*** and ***SG*** stems showed the best antibacterial as well antifungal activities. The reason of this difference compared to PDHPGA from ***SA, SC***, and ***CO*** may be the methylation of the carboxylic moieties of these polymers. As we reported in our previous studies, the same tendency was observed regarding the synthetic oligomeric analogue of PMDHPO, which was the only polymer showing antimicrobial activities against some pathogenic strains at the concentrations that were used [27].

It should be mentioned that, despite there being some references in the literature regarding the antimicrobial activities of catechol-based polymers [7,29], there are no publications concerning sugar-based catechol-containing biopolymers.

### 2.3. Docking Studies

#### 2.3.1. Docking to Antibacterial Targets

Docking studies of several antibacterial targets, such as *E. coli* DNA gyrase, *S. aureus* thymidylate kinase, *E. coli* primase, and *E. coli* MurB, were performed to give a structural insight into their mechanism of action. The docking studies showed that the assessments of the binding free energy to *E. coli* DNA gyrase, thymidylate kinase, *E. coli* primase, and *E. coli* MurA were higher than that to *E. coli* MurB. Therefore, it may be resolved that the inhibition of the *E. coli* MurB enzyme is the most probable mechanism of action of the molecules (Table 4).

**Figure 3:**
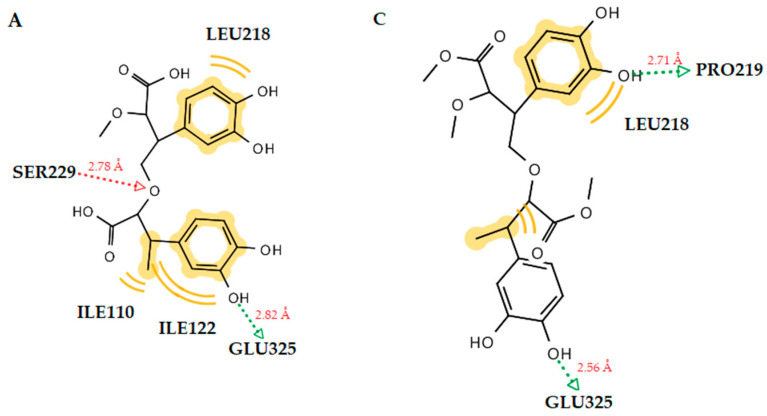

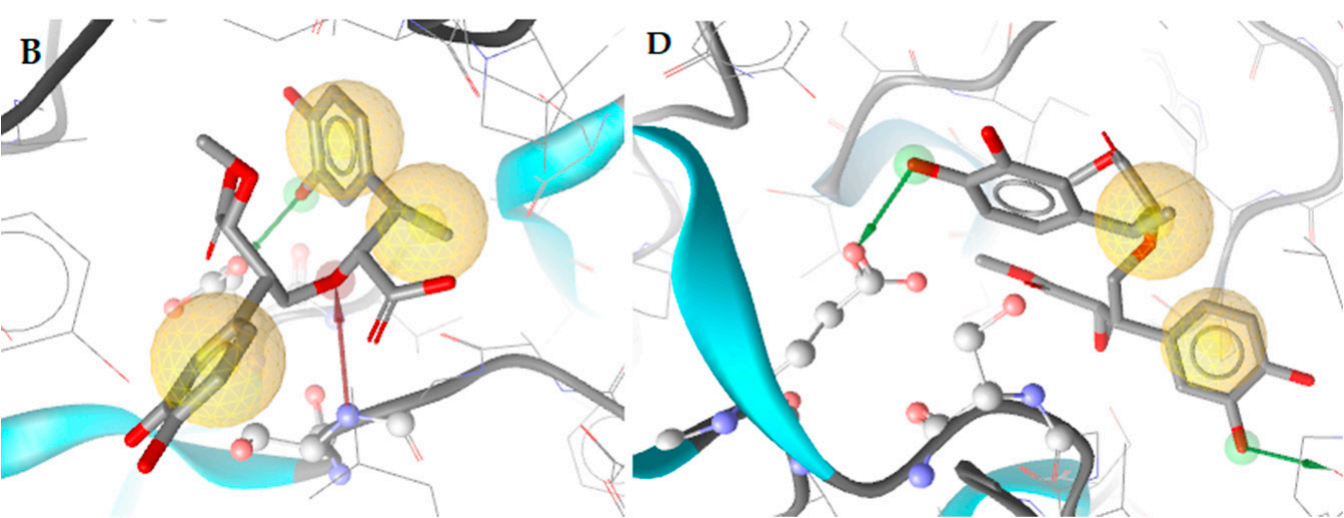
(**A**) A 2D diagram of dimer molecule 1 in *E. coli* MurB. (**B**) Docked pose of dimer molecule **1** in *E. coli* MurB. (**C**) A 2D diagram of dimer molecule **2** in *E. coli* MurB. (**D**) Docked pose of dimer molecule **2** in *E. coli* MurB. Red and green dotted lines show the hydrogen bonds. Yellow spheres represent hydrophobic interactions.

**Figure 4:**
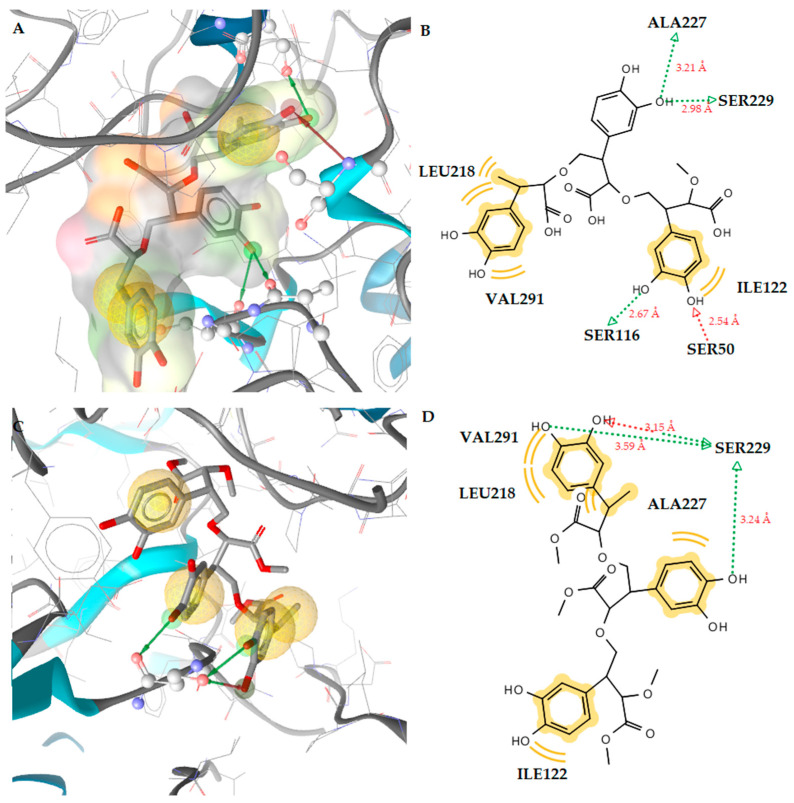
(**A**) Docked pose of trimer molecule **1** in *E. coli* MurB. (**B**) A 2D diagram of trimer molecule **1** in *E. coli* MurB. (**C**) Docked pose of trimer molecule **2** in *E. coli* MurB. (**D**) A 2D diagram of trimer molecule **2** in *E. coli* MurB. Red and green dotted lines show the hydrogen bonds. Yellow spheres represent hydrophobic interactions.

Docking studies of the MurB enzyme revealed that the dimers of molecules **1** and **2** have close free energy of binding values, as do the trimers (Figure 3 and Figure 4). The best docking score was achieved by the trimer of molecule **1**. This molecule showed four favorable hydrogen bond interactions between the hydroxyl substituents and the residues Ser50 (O···H, 2.54Å), Ser116 (O···H, 2.67Å), Ser229 (O···H, 2.98Å), and Ala227 (O···H, 3.21Å). Moreover, hydrophobic interactions were detected between the molecule and the residues Leu218, Ile122, and Val291 (Figure 2). These interactions stabilize the compound–enzyme complex and play a crucial role to the activity of the molecule. Furthermore, the hydrogen bond formation with the residue Ser229 is crucial for the inhibitory activity because this residue takes part in the proton transfer at the second stage of peptidoglycan synthesis [30].

Hydrogen bond interactions with the residue Ser229 were also observed for the trimers of both compounds.

#### 2.3.2. Docking to Antifungal Targets

All the molecules as dimers and trimers, as well as the reference drug ketoconazole, were docked to the lanosterol 14a-demethylase of *C. albicans* and DNA topoisomerase IV, and the results are presented in Table 5.

**Figure 5:**
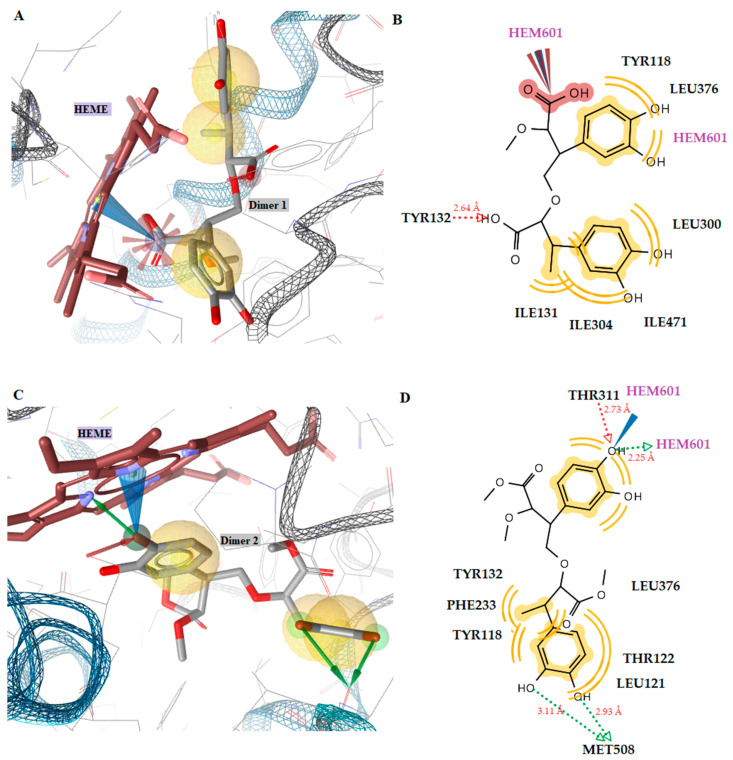
(**A**) Docked pose of dimer molecule **1** in lanosterol 14alpha-demethylase of *C. albicans* (CYP51_ca_). (**B**) A 2D diagram of dimer molecule **1** in CYP51_ca_. (**C**) Docked pose of dimer molecule **2** in CYP51_ca_. (**D**) A 2D diagram of dimer molecule **2** in CYP51_ca_. Red and green dotted lines show the hydrogen bonds. Yellow spheres represent hydrophobic interactions.

**Figure 6:**
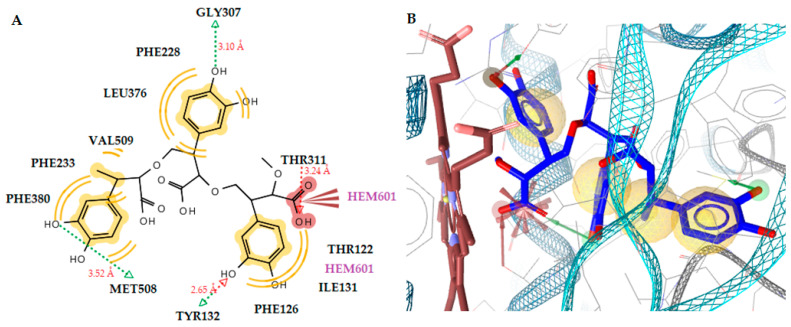
(**A**) A 2D diagram of trimer molecule 1 in CYP51_ca_. (**B**) Docked pose of trimer molecule 1 in CYP51_ca_. Red and green dotted lines show the hydrogen bonds. Yellow spheres represent hydrophobic interactions, and blue arrows represent aromatic interactions.

**Figure 7:**
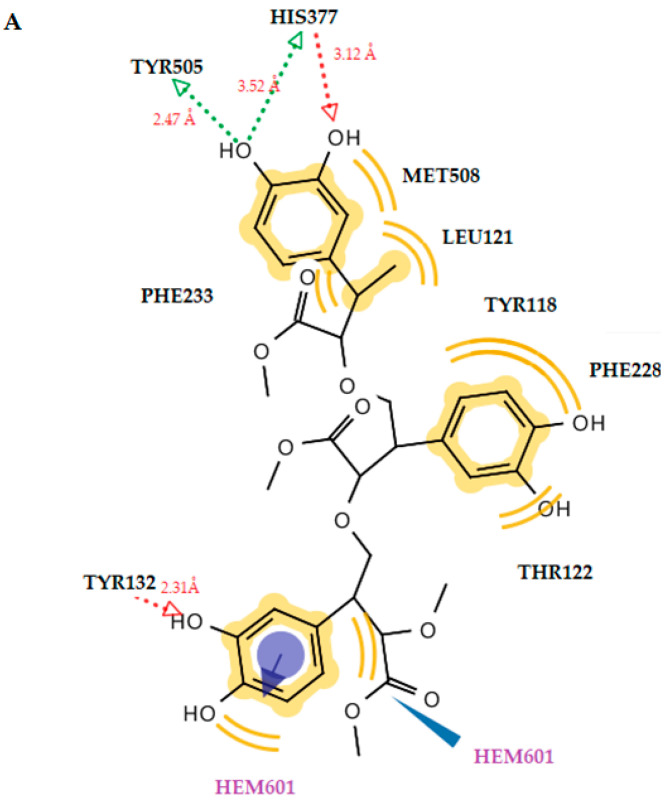

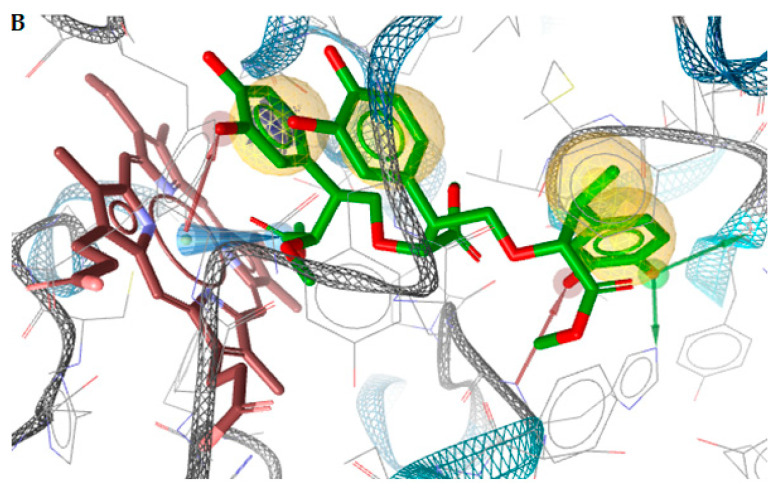
(**A**) A 2D diagram of trimer molecule **2** in CYP51_ca_. (**B**) Docked pose of trimer molecule **2** in CYP51_ca_. Red and green dotted lines show the hydrogen bonds. Yellow spheres represent hydrophobic interactions, and blue arrows represent aromatic interactions.

The docking results showed that molecules **1** and **2**, whether are dimers or trimers, take place inside the active site of the enzyme interacting with the heme group of CYP51_Ca_. A detailed analysis of their binding showed that molecule **1**, in the form of a dimer or trimer, interacts in the same way with the heme group, forming negative ionizable interactions throughout its carboxyl group (Figure 5, Figure 6 and Figure 7). It is noteworthy to mention that, in the case of the dimer of molecule **1**, it interacts with the iron of the heme, making the molecule–enzyme complex more stable and justifying the increased free energy of binding of the trimer.

In the case of molecule **2**, again the dimer suppresses the trimer because it interacts with the heme group, forming a stable hydrogen bond throughout its hydroxyl substituent (Figure 5, Figure 6 and Figure 7). The dimer form of molecule **2** also exhibited the best free energy of binding, with four hydrogen bonds between the hydroxyl substituents and the residues Thr311 (O···H, 2.73Å), Met508 (O···H, 3.93Å), Met508 (O···H, 3.11Å), and the heme group (O···H, 2.25Å). Hydrophobic interactions were also detected with the residues Tyr118, Leu121, Tyr122, Tyr132, Phe233, Thr311, and Leu376. An interaction was also observed between the heme group and the benzene ring of ketoconazole, which formed hydrophobic and aromatic interactions. However, molecule **2** in the dimer forms more and stronger interactions than ketoconazole and a more stable complex of ligand with enzyme. This is probably the reason why this molecule has better antifungal activity than ketoconazole.

### 2.4. Prediction of Toxicity

A prediction of the toxicity profile of the biopolymers was performed using the webserver ProTox-II.

This is a more rapid and less expensive process compared with in vitro testing in animals as well as in vitro testing in cell lines. On the other hand, it allows researchers to greatly reduce the number of animals used in experiments. Several online programs that use in silico methods to access toxicity are available. They predict the average lethal dose, carcinogenicity, mutagenicity, etc.

The PROTOX II program [31] predicts the average lethal dose (LD_50_) in rodents, classifying all chemical compounds into six GHS (Globally Harmonized System of Classification and Labeling of Chemicals) categories [32], depending on the toxicities of the compounds and the LD_50_ values. The LD_50_ values, given in mg/mL, are as follows:

- Class I: fatal if swallowed (LD50 ≤ 5)
- Class II: fatal if swallowed (5 < LD50 ≤ 50)
- Class III: toxic if swallowed (50 < LD50 ≤ 300)
- Class IV: harmful if swallowed (300 < LD50 ≤ 2000)
- Class V: may be harmful if swallowed (2000 < LD50 ≤ 5000)
- Class VI: non-toxic (LD50 > 5000)

The results of the prediction are presented in Table 6. It is worth noting that that the accuracy of the prediction increases as the confidence values increase. In particular, reliable estimates are considered to be more than 0.025.

According to the prediction using the Pro-Tox II webserver, the compounds were found to belong to categories IV and V, with LD50 values ranging from 2000 to 3500 mg/kg and 1000 mg/kg. They are safe to use (Table 6).

## 3. Materials and Methods

### 3.1. Plant Material

Plants were collected from various parts of Georgia province, namely Adjara, Kartli, the surroundings of Tbilisi, from June to September (2010–2020), depending on the climate. Each specimen was labeled, numbered, and annotated with the date of collection and the locality. The plants were identified by the TSMU I. Kutateladze Institute of Pharmacochemistry. Voucher specimens (whole plants) were deposited in the herbarium of the Department of Pharmacobotany of the TSMU Institute of Pharmacochemistry.

### 3.2. Methods

For extraction and isolation, plant material (roots or stems) was cut into small pieces, air-dried, and ground in a mill. Lipids, pigments, and low-molecular-weight compounds (monosaccharides, phenolics, etc.) were removed using Soxhlet extractions with chloroform, methanol, and acetone. The hot-water extraction of pretreated material was followed by dialysis, afforded by crude water-soluble polysaccharides. Further fractionation in a model 8200 stirred ultrafiltration cell (Millipore Corporation, Billerica, MA, USA) on a Biomax-500 ultrafiltration disc (500,000 NMWL) or a Filtron omega series membrane (1 M NMWL), as reported in [33], yielded water-soluble, high-molecular-weight (>500 kDa) preparations. The characterization of PDHPGA performed byUV, IR and NMR spectra of PDHPGA and are given in Supplementary materials Figures S1–S8.

### 3.3. Biological Evaluation

#### 3.3.1. Evaluation of Antimicrobial Activity

Gram-negative bacteria (*Escherichia coli* (ATCC 35210) and *Pseudomonas aeruginosa* (ATCC 27853)) as well as Gram-positive bacteria (*Staphylococcus aureus* (ATCC 6538)) were used. The resistant strains used were methicillin-resistant *S. aureus* (IBRS MRSA 011), resistant *E. coli* (IBRS E003), and resistant *P. aeruginosa* (IBRS P001), which were obtained as described in Kartsev et al. [34]. The organisms were obtained from the Mycological Laboratory, Department of Plant Physiology, Institute for Biological Research ‘Siniša Stanković’, National Institute of Republic of Serbia, Belgrade, Serbia.

#### 3.3.2. Evaluation of Antifungal Activity

The antifungal activities of all investigated samples were tested on strains obtained from the Mycological Laboratory, Department of Plant Physiology, Institute for Biological Research ‘Siniša Stanković’, National Institute of Republic of Serbia, Belgrade, Serbia. *Aspergillus fumigatus* (ATCC 1022), *Aspergillus niger* (ATCC 6275), *Trichoderma viride* (IAM 5061), *Penicillium funiculosum* (ATCC 36839), *P*. *verrucosum var. cyclopium* (*P. v. c*.) (food isolates), and *Candida* albicans (ATCC 10231) were used. The MIC and MBC/MFC concentrations were determined using the modified microdilution method, as previously reported [35,36]. The bacterial/fungal suspensions were adjusted with sterile saline to a concentration of 1.0 × 10^5^ CFU mL^−1^. The compounds were dissolved in a 30% EtOH solution (10 mg mL^−1^) and immediately added into a tryptic soy broth/malt broth medium (100 µL) with a bacterial/fungal inoculum. The microplates were incubated for 24 h at 37 ± 2 °C for bacterial species and for 72 h at 25 ± 2 °C (UE 500, Memmert) for fungi. The results are presented as MICs, MBCs, and MFCs. The MICs obtained from the susceptibility testing of various bacteria/fungi to the tested compounds were also determined using a colorimetric microbial viability assay based on the reduction in INT (p-iodonitrotetrazolium violet) [2-(4-iodophenyl)-3-(4-nitrphenyl)-5-phenyltetrazolium chloride], Merck KGaA, Darmstadt, Germany) color compared with a positive control for each microorganism strain. The MBCs and MFCs were determined using serial sub-cultivations of 2 µL into microtiter plates containing 100 µL of broth per well and further incubation for 24 h. The lowest concentration with no visible growth was defined as the MBC/MFC, indicating 99.5% killing of the original inoculum. The commercial antibiotics ampicillin and streptomycin and the fungicides bifonazole and ketoconazole were used as positive controls, while 30% EtOH was used as a negative control. All experiments were performed in duplicate.

### 3.4. Docking Studies

AutoDock 4.2^®^ software (The Scripps Research Institute, La Jolla, CA, USA) was used for the docking simulation [37]. The binding energies (ΔG) of *E. coli* DNA GyrB, thymidylate kinase, *E. coli* primase, *E. coli* MurB, DNA topoIV, and CYP51 of *C*. *albicans* in complex with the inhibitors were generated using this molecular docking program. The X-ray crystal structure data of all enzymes that were used were obtained from the Protein Data Bank (PDB ID: 1KZN, AQGG, 1DDE, 2Q85, 1S16, and 5V5Z, respectively). All procedures were performed according to our previous paper [35].

## 4. Conclusions

In this work, six plants were collected from various parts of Georgia province, namely Adjara, Kartli, and the surroundings of Tbilisi, from June to September (2010–2020), depending on the climates from which the sugar-based catechol-containing biopolymers from ***SA***, ***SC***, ***SG***, ***CO***, ***BO***, and ***AI*** were extracted. The antimicrobial activities of these biopolymers were evaluated. The study revealed that the antibacterial activities were moderate, and only biopolymers from three plants showed activities against all tested bacteria. Biopolymers from ***CO*** stems as well as ***SC*** and ***AI*** did not show any activities, except low activities against a resistant *P. aeruginosa* strain, which was the most resistant among the three resistant strains.

On the other hand, the antifungal activities were better compared to the antibacterial activities. Biopolymers from ***BO*** stems exhibited the best activities, with MIC/MFC values of 0.37–1.00 mg/mL and 0.75–1.5 mg/L, respectively, followed by those from ***SG*** stems. Biopolymers from ***SC*** and ***AI*** roots showed antifungal activities against all six fungi, in contrast to the antibacterial activities, while biopolymers from ***CO*** stems and ***SA*** roots showed antifungal activities against four fungi and one fungus, respectively. The sugar-based catechol-containing biopolymers from ***BO*** stems demonstrated the best activities among all tested biopolymers against *T. viride*, *P. funiculosum*, *P. cyclpoium var verucosum*, and *C. albicans* (MIC 0.37 mg/mL). In addition, biopolymers from ***SG*** stems were half as active against *A. fumigatus* and *T. viride* as ketoconazole. It is interesting to notice that biopolymers from all plant materials except for ***CO*** stems showed higher potency than ketoconazole against *T. viride.* The biopolymers from all plant materials showed better activities against *C. albicans*, one of the most dreadful fungi, compared to other fungal species. Thus, it can be concluded that plants from Georgia province can be good sources for natural antifungal agents. It should be noticed that, according to prediction results, these biopolymers are not toxic.

## Figures and Tables

**Table 1:** The signal assignments of the solid-state ^13^C{^1^H} CP/MAS NMR spectrum of PMDHPGA (δ, ppm).

С Atom No.	^13^C Chemical Shift, δC, ppm
1	72
2	78
1′	174
(−OCH_3_)	54
1″	130
2″, 5″, 6″	118
3″, 4″	143

**Table 2:** In vitro antibacterial evaluation of PDHPGA and PMDHPO from stems and roots of ***BO***, ***AI***, ***CO***, ***SA***, ***SC***, and ***SG*** (mg/mL).

Compounds		*S. a.*	MRSA	*E. coli*	Rez. *E. coli*	*P. a.*	Rez. *P. a.*
PDHPGA and PMDHPO of ***BO*** (stems)	MIC	1.50	1.50	1.00	1.50	1.00	0.75
	MBC	3.00	3.00	1.50	3.00	1.50	1.50
PDHPGA and PMDHPO of ***AI*** (roots)	MIC	-	-	3.00	-	-	3.00
	MBC	-	-	6.00	-	-	6.00
PDHPGA of ***CO*** (stems)	MIC	-	-	-	-	-	3.00
	MBC	-	-	-	-	-	6.00
PDHPGA of ***SA*** (roots)	MIC	6.00	6.00	6.00	6.00	6.00	3.00
	MBC	9.00	9.00	9.00	9.00	12.00	6.00
PDHPGA of ***SC*** (roots)	MIC	-	-	-	-	-	3.00
	MBC	-	-	-	-	-	6.00
PDHPGA and PMDHPO of ***SG*** (stems)	MIC	0.75	1.00	0.75	1.50	1.00	0.75
	MBC	1.50	1.50	1.50	3.00	1.50	1.50
Ampicillin	MIC	0.10	-	0.15	0.20	0.30	0.20
	MBC	0.15	-	0.20	-	0.50	-
Streptomycin	MIC	0.10	0.10	0.10	0.05	0.10	0.10
	MBC	0.20	-	0.20	0.10	0.20	0.20

*S. a.*—*S. aureus*, MRSA—methicillin-resistant *S. aureus*, *E. c.*—*E. coli*, res. *E. c.*—resistant *E. coli*, *P. a.*—*P. aeruginosa*, res. *P. a.*—resistant *P. aeruginosa*.

**Table 3:** In vitro antifungal evaluation of PDHPGA and PMDHPO from stems and roots of ***BO,*** ***AI***, ***CO***, ***SA***, ***SC***, and ***SG*** (mg/mL).

Compounds		*A. fu.*	*A. n.*	*T. v.*	*P. f.*	*P. v. c.*	*C. a.*
PDHPGA and PMDHPO of ***BO*** (stems)	MIC	0.75	1.00	0.37	0.37	0.37	0.37
	MFC	1.50	1.50	0.75	0.75	0.75	0.75
PDHPGA and PMDHPO of ***AI*** (roots)	MIC	1.50	2.25	0.37	0.75	1.10	0.19
	MFC	3.00	3.00	0.75	1.50	3.00	0.37
PDHPGA of ***CO*** (stems)	MIC	0.75	0.56	0.19	0.37	0.75	0.37
	MFC	1.50	0.75	0.37	0.75	1.50	0.75
PDHPGA of ***SA*** (roots)	MIC	3.00	-	0.75	2.25	2.25	0.09
	MFC	4.50	-	1.50	4.50	4.50	0.19
PDHPGA of ***SC*** (roots)	MIC	0.37	0.37	0.19	0.75	0.75	0.19
	MFC	0.75	0.75	0.37	1.50	1.50	0.37
PDHPGA and PMDHPO of ***SG*** (stems)	MIC	0.37	0.56	0.09	0.56	0.75	0.14
	MFC	0.75	0.75	0.19	0.75	1.50	0.19
Ketoconazole	MIC	0.20	0.20	1.00	0.20	0.20	1.00
	MFC	0.50	0.50	1.50	0.50	0.30	2.00
Bifonazole	MIC	0.15	0.15	0.15	0.20	0.10	0.20
	MFC	0.20	0.20	0.20	0.25	0.20	0.30

*A. f.*—*A. fumigatus*, *A. n.*—*A. niger*, *T. v.*—*T. viride*, *P. f.*—*P. funiculosum*, *P. v. c.*—*P. cyclpoium var verucosum*, *C. a.*—*Candida albicans*.

**Table 4:** Molecular docking binding affinities to antibacterial targets.

No	Est. Binding Energy (kcal/mol)				Residues Involved in Hydrogen Bonds	Residues Involved in Hydrophobic Interactions
	*E. coli* Gyrase 1KZN	*S. aureus* Thymidylate Kinase 4QGG	*E. coli* Primase 1DDE	*E. coli* MurB 2Q85		
**1**	−4.55	−2.56	−2.41	−5.16	Ser229 (O···H, 2.65Å)	Leu218, Ala227, Val291
**1 (dimer)**	−4.31	−2.67	-	−7.80	Ser229 (O···H, 2.78Å), Glu325 (O···H, 2.82Å)	Ile110, Leu218, Ile122
**1 (trimer)**	−2.16	−	−	−9.81	Ser50 (O···H, 2.54Å), Ser116 (O···H, 2.67Å), Ser229 (O···H, 2.98Å), Ala227 (O···H, 3.21Å)	Leu218, Ile122, Val291
**2**	−4.12	−2.46	−2.57	−5.89	Ser229 (O···H, 2.55Å)	Leu218, Ile122
**2 (dimer)**	−3.57	−1.28	−1.06	−6.73	Pro219 (O···H, 2.71Å), Glu325 (O···H, 2.56Å)	Leu218
**2 (trimer)**	−1.67	-	-	−9.16	Ser229 (O···H, 3.15Å), Ser229 (O···H, 3.59Å), Ser229 (O···H, 3.24Å)	Leu218, Ile122, Ala227, Val291

**Table 5:** Molecular docking binding affinities to antifungal targets.

N/N	Est. Binding Energy (kcal/mol)		Residues Involved in Hydrogen Bonds	Residues Involved in Hydrophobic Interactions	Interactions with HEM601
	DNA TopoIV 1S16	CYP51 of *C. albicans* 5V5Z			
**1**	−1.52	−7.96	Tyr132 (O···H, 2.64Å)	Tyr118, Ile131, Tyr132, Leu300, Leu376	Negative ionizable, Hydrophobic
**1 (dimer)**	-	−9.55	Tyr132 (O···H, 2.64Å),	Tyr118, Ile131, Tyr132, Leu300, Leu304, Leu376, Ile407	Negative ionizable, Hydrophobic, Fe-binding
**1 (trimer)**	-	−9.13	Tyr132 (O···H, 2.65Å), Gly307 (O···H, 3.10Å), Thr311 (O···H, 3.24Å), Met508 (O···H, 3.52Å)	Ile131, Thr122, Phe126, Phe380, Phe233, Phe228, Leu376, Val509, Met508	Negative ionizable, Hydrophobic
**2**	−1.78	−9.26	Tyr132 (O···H, 2.67Å)	Tyr118, Leu121, Tyr122, Tyr132, Phe233, Thr311, Leu376	Negative ionizable, Hydrophobic
**2 (dimer)**	−1.78	−11.35	Thr311 (O···H, 2.73Å), Met508 (O···H, 3.93Å), Met508 (O···H, 3.11Å), Hem601 (O···H, 2.25Å)	Tyr118, Leu121, Tyr122, Tyr132, Phe233, Thr311, Leu376	Hydrophobic, H-Bond, Fe-binding
**2 (trimer)**	-	−11.10	Tyr132 (O···H, 2.31Å), His377 (O···H, 3.12Å), His377 (O···H, 3.52Å), Tyr505 (O···H, 2.47Å)	Met508, Leu121, Tyr118, Phe228, Thr122, Phe233	Hydrophobic, aromatic, Fe-binding
**ketoconazole**	-	−8.23	Tyr64 (O···H, 2.51Å)	Tyr118, Ile131, Tyr132, Leu300, Ile304, Leu376, Met508	Hydrophobic, aromatic

**Table 6:** Prediction of toxicity.

No	Predicted LD50 (mg/kg)	Predicted Toxicity Class	Hepatotoxicity	Carcinogenicity	Immunotoxicity	Mutagenicity	Cytotoxicity
**1**	2000	IV	Inactive (0.6)	Inactive (0.56)	Inactive (0.54)	Inactive (0.94)	Inactive (0.95)
**2**	2000	IV	Inactive (0.57)	Inactive (0.56)	Inactive (0.62)	Inactive (0.91)	Inactive (0.92)
**1 (dimer)**	3500	V	Inactive (0.79)	Inactive (0.73)	Inactive (0.58)	Inactive (0.77)	Inactive (0.86)
**2 (dimer)**	3500	V	Inactive (0.80)	Inactive (0.74)	Inactive (0.50)	Inactive (0.78)	Inactive (0.84)

Numbers in brackets indicate possibilities.

## Data Availability

Not applicable.

## Supplementary Materials

The following supporting information can be downloaded at https://www.mdpi.com/article/10.3390/antibiotics12020285/s1, Table S1. Signals assignments of the ^13^C and ^1^H NMR spectra of PDHPGA-SA, PDHPGA-SC, PDHPGA-CO, PDHPGA-SG, PDHPGA-AI and PDHPGA-BO; Figure S1. UV spectra (H_2_O, λ_max_, nm) of PDHPGA-SA, PDHPGA-SC, PDHPGA-SG, PDHPGA-AI, PDHPGA-CO and PDHPGA-BO; Figure S2. The IR spectra of PDHPGA-SA, PDHPGA-SC, PDHPGA-SG, PDHPGA-AI, PDHPGA-CO and PDHPGA-BO; Figure S3. The ^13^C NMR spectrum of PDHPGA-SA, PDHPGA-SC and PDHPGA-CO; Figure S4. The ^1^H NMR spectrum of PDHPGA-SA, PDHPGA-SC and PDHPGA-CO; Figure S5. The HSQC spectrum of PDHPGA-SA, PDHPGA-SC and PDHPGA-CO; Figure S6. The ^13^C NMR spectrum methylated in carboxylic groups of PDHPGA-SG, PDHPGA-AI and PDHPGA-BO; Figure S7. The ^1^H NMR spectrum of methylated in carboxylic groups of PDHPGA-SG, PDHPGA-AI and PDHPGA-BO; Figure S8. The 2D DOSY spectrum of PDHPGA (R=H) and methylated in carboxylic groups of PDHPGA (R=CH_3_)**.**
