# Supplementary material for: Antimicrobial Activity of Catechol-Containing Biopolymer Poly[3-(3,4-dihydroxyphenyl)glyceric Acid] from Different Medicinal Plants of Boraginaceae Family
Source: Antibiotics (Basel). 2023 Feb 1;12(2):285. doi: 10.3390/antibiotics12020285 (PMC9952037; doi:10.3390/antibiotics12020285)
Supplement: Supplementary file 1 [file antibiotics-12-00285-s001.zip › antibiotics-2079230-supplementary.pdf]

Supplementary materials

UV spectra were recorded on a Hitachi 150-20 spectrophotometer. IR spectra were registered in KBr pellets on a Perkin-Elmer 571 spectrophotometer. NMR spectra were taken on a Bruker DRX-500 spectrometer for 1% solutions of the polymers in D_2O at 70-80ºC using acetone (δH 2.225 ppm, δC 31.45 ppm) as the internal standard (sealed tubes).

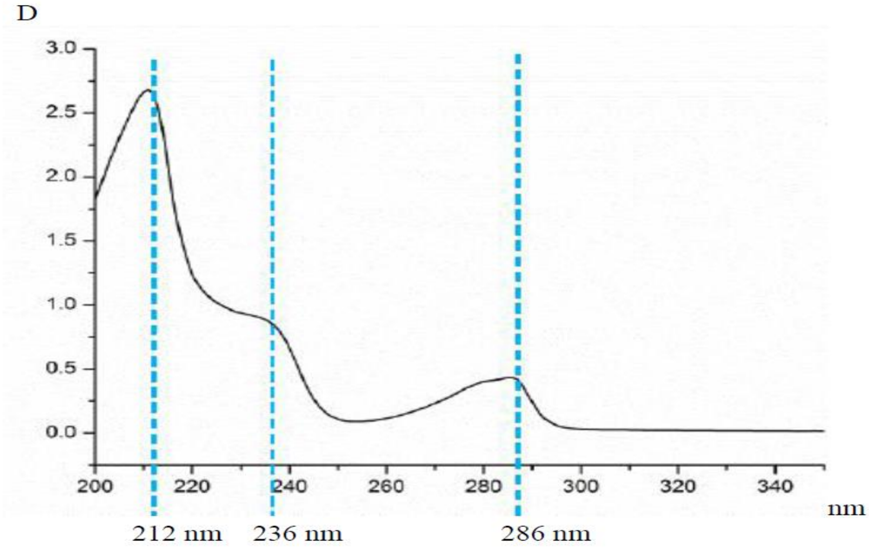

Figure S1. UV spectra (H_2O, λ_{max}, nm) of PDHPGA-SA, PDHPGA-SC, PDHPGA-SG, PDHPGA-AI, PDHPGA-CO and PDHPGA-BO

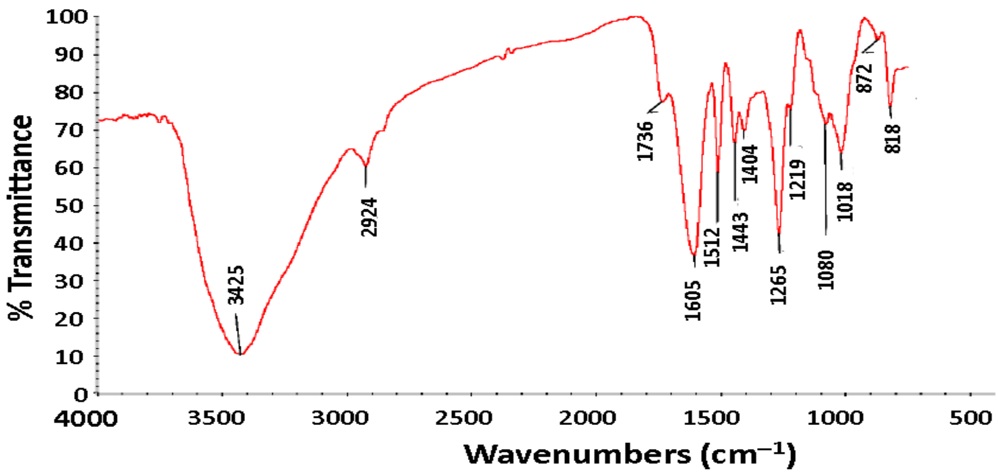

Figure S2. The IR spectra of PDHPGA-SA, PDHPGA-SC, PDHPGA-SG, PDHPGA-AI, PDHPGA-CO and PDHPGA-BO.

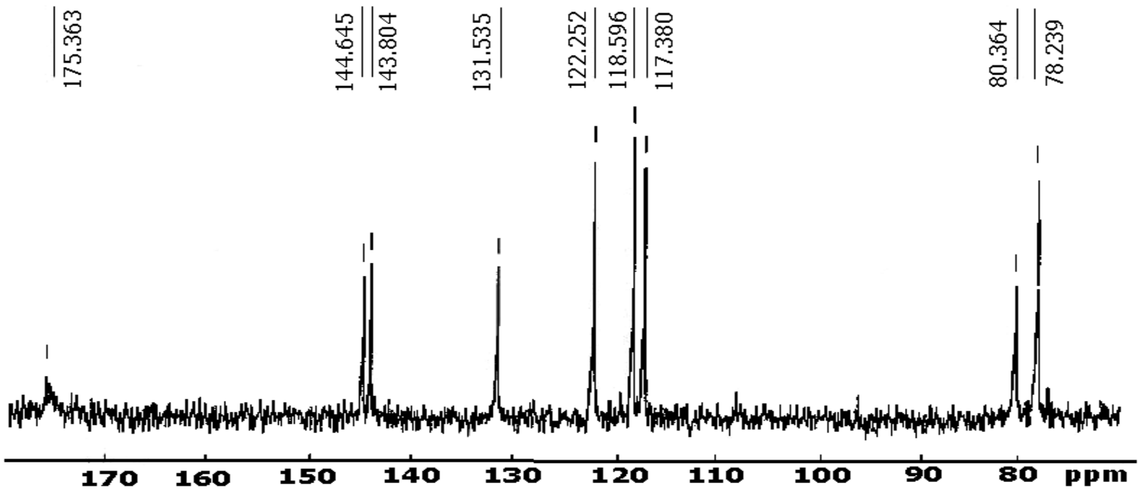

Figure S3. The ^{13}C NMR spectrum of PDHPGA-SA, PDHPGA-SC and PDHPGA-CO.

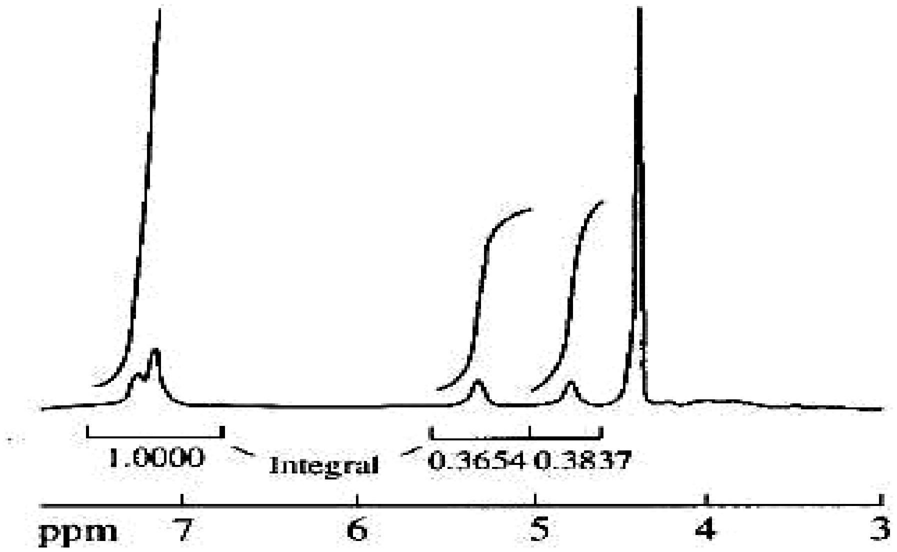

Figure S4. The ^{1}H NMR spectrum of PDHPGA-SA, PDHPGA-SC and PDHPGA-CO.

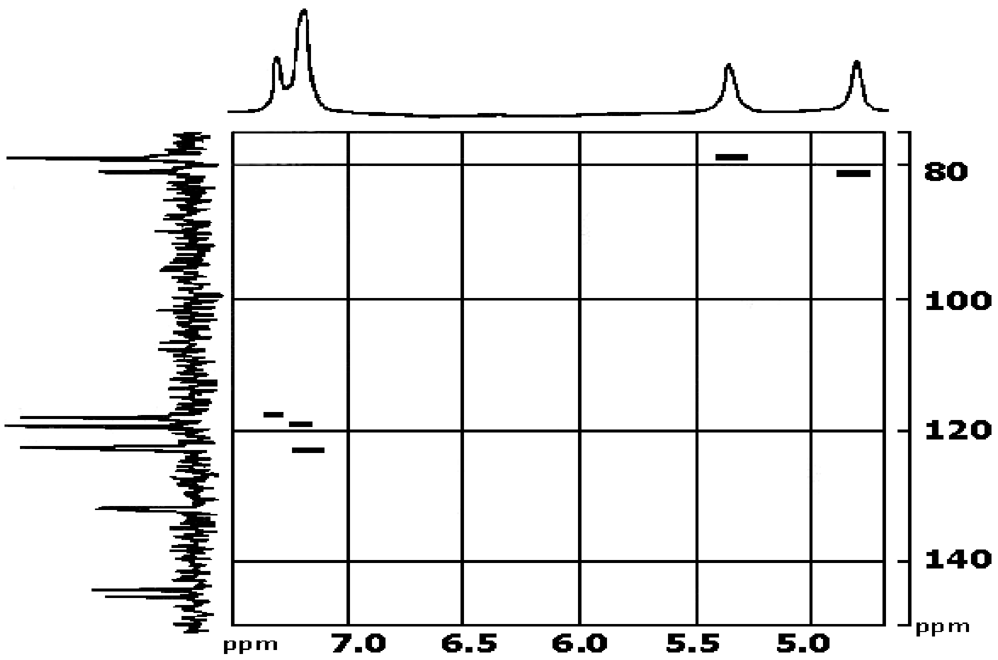

Figure S5. The HSQC spectrum of PDHPGA-SA, PDHPGA-SC and PDHPGA-CO.

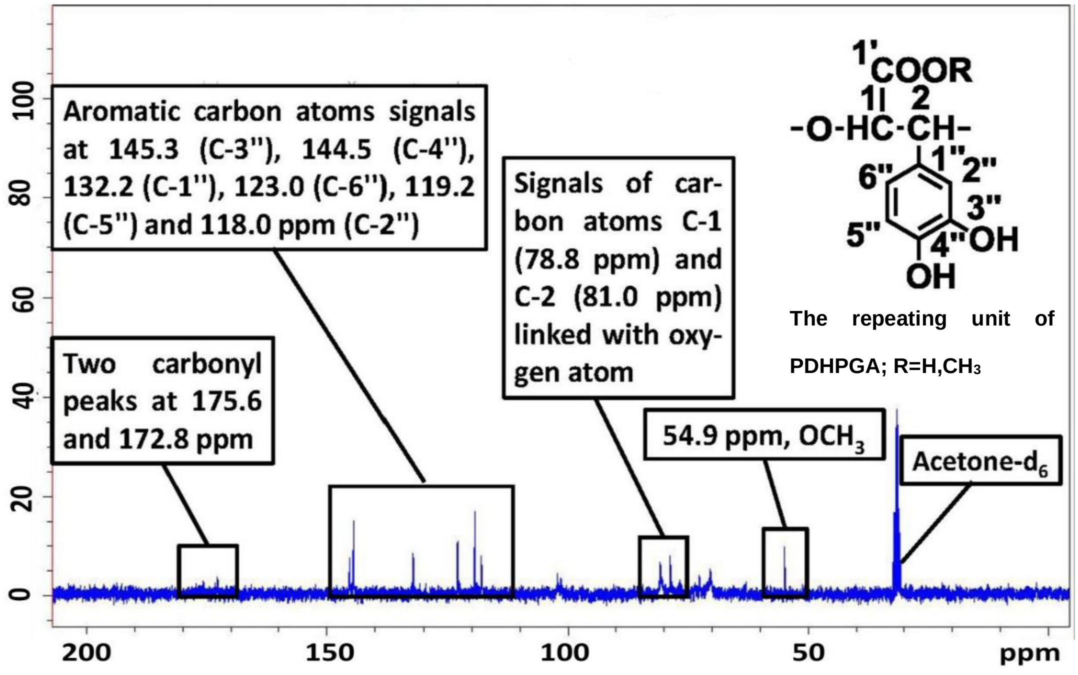

Figure S6. The ¹³C NMR spectrum methylated in carboxylic groups of PDHPGA-SG, PDHPGA-AI and PDHPGA-BO.

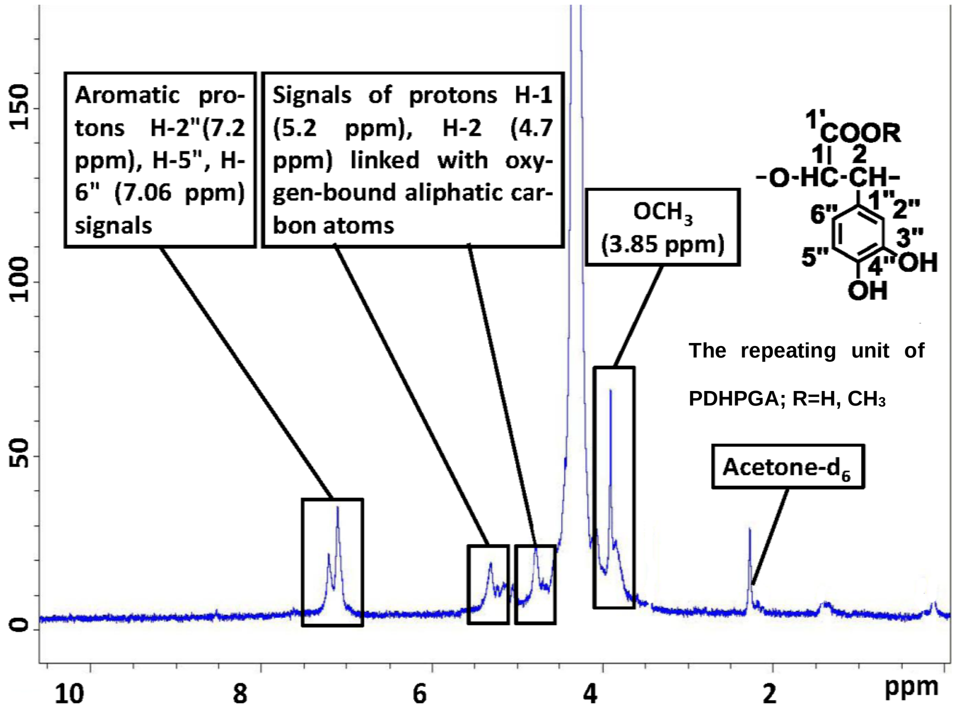

Figure S7. The ^{1}H NMR spectrum of methylated in carboxylic groups of PDHPGA-SG, PDHPGA-AI and PDHPGA-BO.

Table S1. Signals assignments of the ^{13}C and ^{1}H NMR spectra of PDHPGA-SA, PDHPGA-SC, PDHPGA-CO, PDHPGA-SG, PDHPGA-AI and PDHPGA-BO

	C atom no.	^{13}C chemical shift, δC, ppm	^{1}H chemical shift, δH, ppm
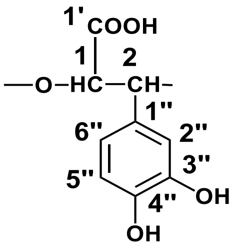	1'	175.4	
	1	78.2	5.3
	2	80.4	4.9
	1''	131.5	
The repeating unit of PDHPGA.	2''	117.4	7.2
	3''	144.7	
	4''	143.8	
	5''	118.6	7.1
	6''	122.3	7.1
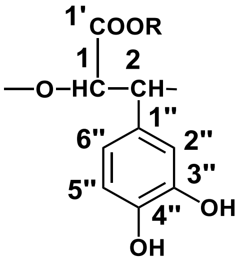	1'	175.6 (COO–)<br>172.8 ($COOCH_3$)<br>54.9 (OCH_3)	3.85 (OCH_3)
The repeating unit of PDHPGA (R=H) and methylated in carboxylic groups of PDHPGA ($R=CH_3$).	1	78.8	5.2
	2	81.0	4.7
	1''	132.2	
	2''	118.0	7.2
	3''	145.3	
	4''	144.5	
	5''	119.2	7.1
	6''	123.0	7.1

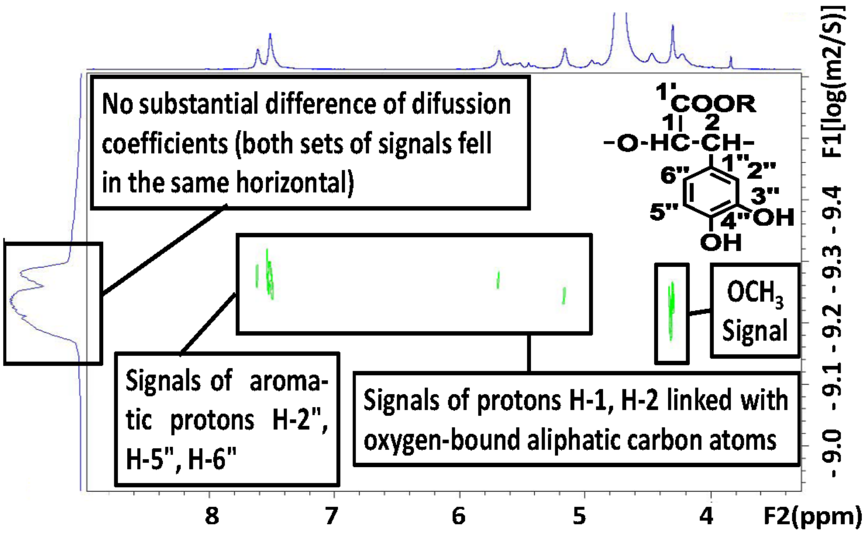

Figure S8. The 2D DOSY spectrum of PDHPGA (R=H) and methylated in carboxylic groups of PDHPGA (R=CH_3)
